# Assessing the therapeutic potential of a panel of novel VCAM-1 antibodies using microfluidic and three-dimensional *in vitro* models of vascular inflammation

**DOI:** 10.1093/abt/tbaf025

**Published:** 2025-11-04

**Authors:** Jessica R Pickett, Lucia F Zacchi, Binura Perera, Yuao Wu, Hang Thu Ta

**Affiliations:** School of Environment and Science, Griffith University, 170 Kessels Road, Nathan, Queensland 4111, Australia; Queensland Quantum and Advanced Technologies Research Institute, Griffith University, 170 Kessels Road, Nathan, Queensland 4111, Australia; School of Chemistry and Molecular Biosciences, The University of Queensland, 280-284 Sir Fred Schonell Drive, St Lucia, Queensland 4072, Australia; Australian Institute for Bioengineering and Nanotechnology, The University of Queensland, 280-284 Sir Fred Schonell Drive, St Lucia, Queensland 4072, Australia; School of Environment and Science, Griffith University, 170 Kessels Road, Nathan, Queensland 4111, Australia; School of Environment and Science, Griffith University, 170 Kessels Road, Nathan, Queensland 4111, Australia; School of Environment and Science, Griffith University, 170 Kessels Road, Nathan, Queensland 4111, Australia; Queensland Quantum and Advanced Technologies Research Institute, Griffith University, 170 Kessels Road, Nathan, Queensland 4111, Australia

**Keywords:** vascular cell adhesion molecule, VCAM-1, antibody therapy, atherosclerosis, monocyte, endothelial cell

## Abstract

**Objective:**

Antibodies against vascular cell adhesion molecule (VCAM)-1 represent an attractive strategy for atherosclerosis and cardiovascular disease management due to their ability to selectively block leukocyte-endothelial interactions involved in inflammatory cell recruitment. Herein, seven novel anti-VCAM-1 monoclonal antibodies (mAbs) generated from phage display biopanning were tested using a series of *in vitro* models of cell recruitment to determine their potential utility for treating atherosclerosis.

**Methods and Results:**

We assessed the inhibitory effects of the test antibodies on cell adhesion and transmigration using a series of *in vitro* assays that incorporated three-dimensional microfluidics and collagen hydrogel models. In summary, each of our mAb candidates were found to reduce RAW264.7 monocyte adhesion to activated SVEC4–10 endothelial monolayers under static conditions. Subsequently, the three most effective candidates from this assay—2E2, 3C12, and 3H4—were shown to inhibit monocyte adhesion to endothelial microvessels under flow conditions and monocyte transmigration into endothelialized gel matrices under static conditions.

**Conclusion:**

These results indicate that our novel anti-VCAM-1 mAbs can effectively inhibit monocyte adhesion and transmigration *in vitro*, supporting the therapeutic rationale of VCAM-1 immunoblockade for the targeted treatment of atherosclerosis.

## Teaser

**Statement of Significance** This study provides valuable insights for antibody drug discovery technologies and pre-clinical models of cardiovascular diseases.

## Graphical Abstract

**Graphical Abstract:**
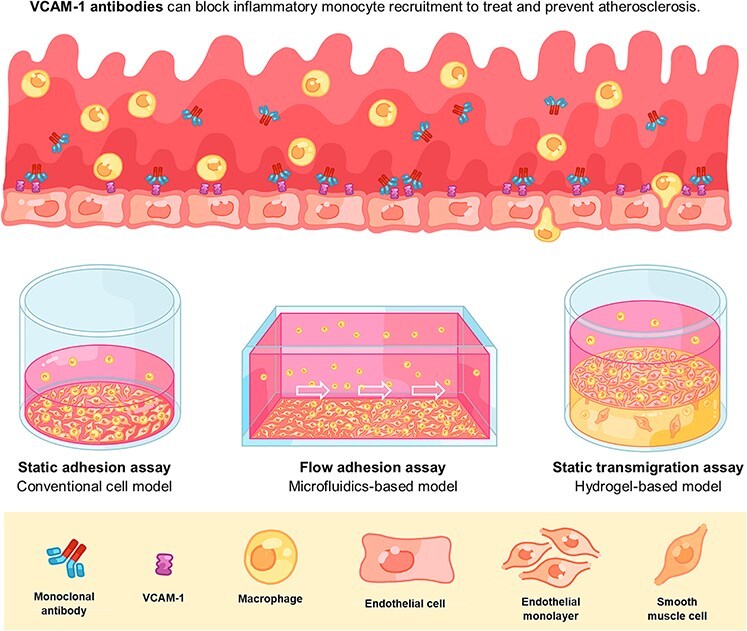

## Introduction

Atherosclerosis is a degenerative vascular condition that involves the progressive build-up of lipid-laden plaque (also known as ‘atheroma’) in inflamed arteries, eventually resulting in narrowed vessels that obstruct normal blood flow [1]. One of the most dangerous aspects of the disease is its insidious nature, often completely evading clinical detection until it has already culminated in life-threatening complications such as myocardial infarction or ischaemic stroke [2]. Traditionally, the treatment and prevention of atherosclerosis-related cardiovascular diseases (CVDs) has been focused primarily on managing classical cardiac risk factors, such as hypertension and hypercholesterolaemia [3]. However, despite the efficacy of existing pharmacological interventions in controlling these established health risks, CVD remains the leading cause of mortality globally [4]. More recently, the scientific understanding of atherosclerosis has evolved to recognize the role of the innate immune response– particularly of dysregulated monocyte recruitment –in the development and progression of atherosclerotic lesions [5]. As such, there has been a notable shift in research attention towards novel therapies that focus on directly countering the inflammatory pathogenesis of atherosclerotic lesion formation [6].

Therapeutic antibodies that selectively target cell adhesion and recruitment at atheromatous lesions and lesion-predisposed regions present an attractive avenue for anti-atherosclerotic drug development [7]. Cell adhesion molecules (CAMs) facilitate the attachment and transmigration of monocytes across the vessel wall into the subendothelial space before they transform into the lipid-engorged foam cells that constitute the atherosclerotic plaque [1]. Naturally, several CAM-inhibiting agents, including high-affinity antibodies, have been investigated as novel therapeutics for atherosclerosis [7]. In particular, antibodies against vascular cell adhesion molecule (VCAM)-1 have attracted significant research attention for their capacity to selectively bind to inflamed regions of the vascular endothelium during atherosclerosis while avoiding adjacent, non-inflamed tissues [8]. However, the therapeutic potential of anti-VCAM-1 antibodies for treating atherosclerotic lesions has been somewhat overshadowed by their usefulness as targeting ligands in vectorized drug delivery systems [9]. Currently, a study by Park *et al.* [10] assessing the effects of novel anti-VCAM-1 antibodies in hypercholesterolemic mouse models remains the most comprehensive investigation of the therapeutic potential of VCAM-1 antibodies for treating atherosclerosis.

Current advancements in antibody discovery technologies have allowed researchers to generate a vast arsenal of novel anti-VCAM-1 antibodies that may also be suitable as future drug candidates [8, 11]. Though the most extensively studied anti-VCAM-1 antibody candidates tend to target the first and second domains of the protein, researchers have more recently begun to investigate the effects of targeting adjacent and alternative domains on receptor binding activity and signalling function [10–12]. Therefore, in optimizing the production pipeline of new antibodies for therapeutic use, improving disease modelling platforms for pre-clinical drug testing has become increasingly necessary. The limitations of conventional cell-based models to simulate the physiological conditions of the vascular environment have fuelled the demand for improved *in vitro* technologies that can serve as better platforms for disease modelling and drug testing. In particular, microfluidic devices incorporating pulsatile flow and three-dimensional (3D) cell culture models that simulate the layered vascular architecture are the predominant approaches for improving *in vitro* modelling of atherosclerosis [13]. Implementing these techniques allows researchers to better replicate biological processes involved in atherosclerotic lesion formation, such as inflammatory cell recruitment, to reliably examine the therapeutic mechanisms and efficacy of novel drug candidates.

This study employs a series of *in vitro* assays modelling pathological monocyte recruitment during vascular inflammation to preliminarily evaluate the therapeutic potential of seven novel anti-VCAM-1 monoclonal antibodies (mAbs) as possible drug candidates for atherosclerosis. In our recent work (Perera *et al.* [11]), these antibody candidates—1A9, 2D3, 2D8, 2E2, 2E6, 3C12, and 3H4—identified by phage display biopanning and in scFv format, were shown to reduce monocyte adhesion to activated endothelial monolayers *in vitro* (with the exception of clone 3C12, which was not tested). However, monocyte recruitment involves several molecular processes outside of leukocyte-endothelial attachment and, thus, further experimentation is required to reliably predict the effects of our test antibodies in the human vascular environment. To achieve this, we employed a microfluidics-based microvessel model to measure antibody inhibition of cell adhesion under human physiological flow conditions and a 3D intimal model to assess cell transmigration under static conditions using murine cell lines. This research holds considerable implications for the medical application of VCAM-1 mAbs for the targeted treatment of atherosclerotic lesions. Moreover, this study also validates the utility of microfluidics- and hydrogel-based atherosclerosis models for the *in vitro* testing of novel therapeutics targeting cell recruitment.

## Materials and method

The full description of materials and methods is provided in Supporting Information.

### Development and selection of anti-VCAM-1 mAbs

Before the commencement of this project, a panel of anti-VCAM-1 mAbs (1A9, 2D3, 2D8, 2E2, 2E6, 3C12, and 3H4) with binding affinity to mouse VCAM-1 was generated. Phage display biopanning was used to screen a human naïve single-chain variable fragment (scFv) library for clones specifically binding mouse VCAM-1. The scFvs were reformatted into full-length, mouse immunoglobulin G (IgG)_2a_ mAbs and expressed in mammalian cells following standard protocols [11, 14]. The known binding specificities of the scFvs for each of the seven IgG domains of VCAM-1 are listed in Table 1 (with further information in Supplementary Fig. S1).

**Table 1:** List of the Ig-like domain binding specificities of the seven novel anti-VCAM-1 antibody candidates and the positive control anti-VCAM-1 antibody used in this study

Antibody name	RRID	Domain specificity for VCAM-1 protein
429 (MVCAM)	AB_467419	Domain 1
1A9	–	Domain 3
2D3	–	Domain 2
2D8	–	Domain 3
2E2	–	Domain 3
2E6	–	Domain 5
3C12	–	Domain 1
3H4	–	Domain 2

### Other antibodies

Other antibodies used in this study are as follows. Rat anti-mouse anti-VCAM-1 (CD106) 429 mAb (Thermo Fisher Scientific #14–1061-82, RRID: AB_467419), rat anti-mouse VE-cadherin (CD144, Alexa Fluor® 488) BV13 mAb (Thermo Fisher Scientific #53–1441-80, RRID: AB_1210528), and mouse IgG_2a_ isotype control (Thermo Fisher Scientific #02–6502, RRID: AB_2532951) were purchased from Thermo Fisher Scientific.

### Static monocyte adhesion assay on the microplate

The static monocyte-endothelial cell adhesion assay was adapted from Park *et al*. [10] SVEC cells were seeded in 96-well plates and cultured in DMEM for 24 h, then stimulated with 100 ng/ml LPS for another 24 h. Cells were pre-treated with 20 μg/ml anti-VCAM-1 antibodies (including positive control, isotype control, and test clones) for 1 h. Meanwhile, RAW monocytes were labelled with 50 ng/ml DiOC_6_, washed, and added to the wells (1.0 × 10^4^ cells/well), followed by a 10 min incubation at 37°C in the dark. Wells were then washed to remove unbound cells and fixed with 4% PFA.

### Fluorescence spectrophotometry and microscopy of static binding assay

Monocyte adhesion was quantified using two methods: fluorescence plate reader analysis and fluorescence microscopy (full details are provided in the Supporting Information). Cumulative fluorescence from DiOC_6_-labelled monocytes was measured and normalized to control wells. Microscopy images were analyzed using Fiji software to count adherent cells, providing an average cell density per unit area (mm^2^) [15].

### Microfluidics chip fabrication

Microfluidics chips were fabricated according to standard photolithography and polydimethylsiloxane (PDMS) soft lithography techniques [16]. The PDMS mixture was cast onto a silicon mould, cured, and bonded to glass slides via oxygen plasma treatment. The final device featured a single rectangular microchannel with defined inlet and outlet ports, suitable for cell culture and flow-based assays. For this study, the design of the microfluidic device consisted of a single rectangular microchannel chamber (255 μm wide and 100 μm high) with circular inlet and outlet ports (100 μm in diameter).

### Microvessel formation in the microfluidics device

Endothelial cells were seeded within the microfluidic devices using a protocol previously reported by Akther *et al.* [17] Briefly, endothelial cells were seeded into sterilized, collagen-coated microfluidic devices [18] to promote adhesion. A high-density SVEC cell suspension was injected, followed by incubation under oscillatory flow and standard culture conditions to form a uniform 3D endothelial microvessel. The device was connected to a peristaltic pump and maintained under physiological flow rates, starting low (12 μl/min) and increasing to arterial shear conditions (31 μl/min) over 24 h to support vessel formation. This flow rate was calculated to equate to typical arterial shear rate (1000 s^−1^) based on the dimensions of the microchannel and Newton’s Law of Viscosity [19]. All treatments were subsequently perfused at this flow rate.

### Flow-based monocyte adhesion assay on the microfluidic chip

After forming a confluent endothelial monolayer, microvessels were stimulated with 1 μg/ml LPS for 8 h, followed by a 2 h pre-treatment with 20 μg/ml anti-VCAM-1 antibodies. Fluorescently labelled RAW monocytes were then perfused through the microchannel to assess monocyte rolling and adhesion under flow using fluorescence microscopy. Adhesion was quantified by counting Hoechst-labelled cells per mm^2^ from three randomly selected fields within the microchannel at 20× magnification (0.5 mm^2^/field) using Fiji software.

### Formation of cell-hydrogel constructs for 3D cell culture

Cell-hydrogel constructs were prepared using collagen I and MOVAS smooth muscle cells to model the subendothelial environment. The collagen and smooth muscle cell components of the resultant hydrogel matrices served to model the extracellular microenvironment of the vascular subendothelial space [20]. After gelation in 96-well plates, constructs were endothelialized with SVEC cells and cultured for 5 days to form a confluent monolayer [21]. Endothelial barrier integrity was confirmed via VE-cadherin immunostaining and assessed by brightfield and fluorescence microscopy.

### Monocyte transmigration assay under static, non-flow conditions

Once endothelial monolayers reached confluency, constructs were stimulated with 100 ng/ml LPS for 24 h, followed by a 1 h pre-treatment with 20 μg/ml anti-VCAM-1 antibodies. RAW monocytes were then added and incubated for 1 h to allow adhesion. Non-adherent cells were collected and counted using a haemocytometer. Constructs were then replenished with fresh media and incubated for 24 h to allow cell migration before further analysis.

### Visual assessment of monocyte transmigration by Giemsa stain microscopy

To qualitatively assess monocyte transmigration, cell-hydrogel constructs were stained with Giemsa, which selectively labels leukocytes [22, 23]. After fixation, standard staining and washing steps were followed. Gels were then placed on coverslips for brightfield microscopy, and monocytes were counted on both the endothelial surface and within the hydrogel matrix from randomly selected fields within the microplate wells at 20× magnification (0.5 mm^2^/field).

### Quantitative measurement of transmigration by Giemsa smear

To quantify monocyte adhesion and transmigration, an adapted Giemsa smear method was used [24]. Non-transmigrated monocytes were detached from the hydrogel surface using trypsin, collected, and stained for counting *via* Giemsa smear. Transmigrated cells were isolated by enzymatic digestion of the gel with collagenase, followed by centrifugation and staining using the same smear protocol. Cells were visualized and counted under brightfield microscopy to assess antibody efficacy.

### Statistical analysis

Data are presented as mean ± standard deviation, with statistical significance indicated by ^*^P < .05, ^**^P < .01, ^***^P < .005, ^**^P < .001. Group comparisons were performed using one-way ANOVA with Tukey’s post-hoc testing. All analyses were conducted using GraphPad Prism 10. The total number of experimental replicates (N), which includes both independent experiments and technical replicates, are indicated in the figure legends for each experiment.

## Results

### Novel VCAM-1 antibodies decreased monocyte attachment to inflamed endothelial monolayers under static conditions

Prior to this study, Perera *et al.* [11] generated a panel of novel anti-VCAM-1 antibodies by phage display biopanning of a human naïve scFv library. In summary, a pool of seven novel mAb candidates—1A9, 2D3, 2D8, 2E2, 2E6, 3C12, and 3H4—was selected based on their binding to seven-domain, murine VCAM-1. As part of the study, the scFv antibody fragments (with the exception of 3C12, which was not included) were tested for their capacities to inhibit monocyte-endothelial adhesion, which is a critical process in vascular inflammation. All the tested candidates were able to significantly reduce monocyte attachment to activated endothelial cells, except for 2E6 (*P* = .056) [11]. These scFvs were reformatted into full-length, mouse IgG_2a_ mAbs [11] and used in this study.

**Figure 1:**
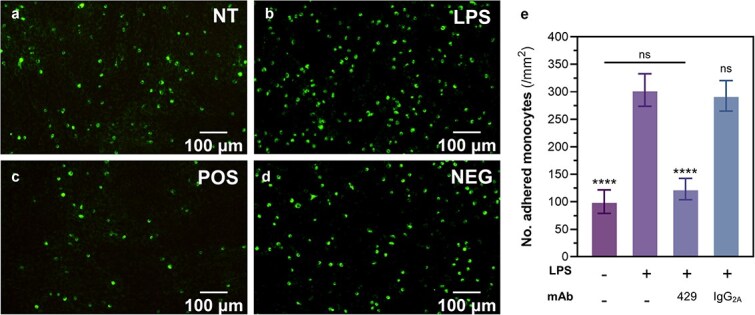
Preliminary adhesion assay assessing the effects of LPS stimulation and VCAM-1 antibody blockade on adhesive monocyte-endothelial interactions. (a-d) Representative fluorescent microscopy (20×) images of labelled RAW264.7 monocytes attached to SVEC4-10 endothelial monolayers pre-treated with (a) non-supplemented cell culture media, (b) LPS, (c) LPS followed by anti-VCAM-1 429 mAb treatment, and (d) LPS stimulation followed by a non-VCAM-1 binder treatment as negative control (mouse IgG2a isotype control antibody). (e) Graphical comparison of cell adhesion with LPS stimulation and anti-VCAM-1 antibody blockade. Cell adhesion was quantified by counting labelled cells in 3 random fields of view observed at 20× magnification, then calculating the number of adhered monocytes per mm2. The graph shows the mean +/- SD of 4 independent experiments with 2 sample replicates each (total N = 8) analyzed by ANOVA and post-hoc Tukey testing. Asterisk labels indicate statistical significance of the non-treated control and mAb treatment groups compared to the LPS-stimulated group unless otherwise specified. ns *p* > 0.05, ^*^p < 0.05, ^**^p < 0.01, ^***^p < 0.001, ^****^p < 0.0001.

To validate and compare the immunoblockade effects of the selected full-length mAbs on VCAM-1-mediated cell-to-cell interactions, we performed conventional cell adhesion assays quantifying monocyte attachment to endothelial monolayers *in vitro*. As a proof-of-concept, a precursory cell adhesion assay was performed to verify the effects of inflammatory LPS stimulation and VCAM-1 immunoblockade on monocyte-endothelial interactions. Briefly, confluent endothelial monolayers were pre-treated or not with LPS, and LPS-treated samples were also treated with culture media alone (no antibody), anti-VCAM-1429 mAb, or a non-VCAM-1-binding negative control for 1 h and then incubated with suspensions of fluorescent monocytes for 10 min before rinsing with DPBS. As summarized in (Fig. 1e), this assay demonstrated that inflammatory stimulation by LPS treatment (Fig. 1b) significantly increased (*P* < .001) monocyte attachment compared to the non-treated control group (Fig. 1a). These observations are corroborated by previous studies demonstrating the stimulatory effects of LPS induction on monocyte-endothelial interactions [25, 26]. Moreover, preincubation of cell monolayers with positive control anti-VCAM-1429 mAb (Fig. 1c) after LPS stimulation was shown to significantly reduce (*P* < .001) the mean level of monocyte adhesion to a level that was comparable to that observed with the non-stimulated endothelia (Fig. 1a). This ability of the 429 mAb to inhibit adhesion of RAW264.7 monocytes to SVEC4–10 monolayers was similar to that of two novel anti-VCAM-1 mAbs (H6 and 7H) tested previously by Park *et al.* [10] to inhibit the adhesion of U937 monocytes to tumour necrosis factor-α–stimulated HUVEC monolayers. Further, the negative control (mouse IgG_2a_ isotype control, a non-VCAM-1 antibody binder) was unable to reduce monocyte adhesion to SVEC4–10 cells upon LPS stimulation (Fig. 1d), indicating that the impact of the 429 antibody treatment on adhesion is due to VCAM-1 blockade.

**Figure 2:**
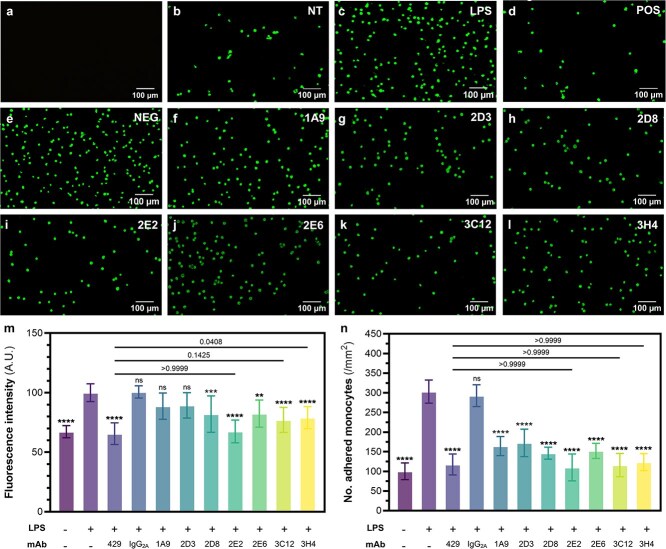
Competitive adhesion assay assessing the potential therapeutic capabilities of a panel of novel anti-VCAM-1 mAb candidates to inhibit monocyte-endothelial interactions under static conditions. (a-k) Representative fluorescent microscopy (20×) images of labelled RAW264.7 monocytes attached to SVEC4-10 endothelial monolayers. This includes a (a) blank control of endothelial monolayers receiving no fluorescent monocyte treatment. For samples that were subjected to monocyte treatment, cell monolayers were pre-incubated with (b) non-supplemented cell culture media, with (c) LPS, or LPS stimulation followed by treatment with (d) commercial anti-VCAM-1 429 mAb (positive control), (e) negative mouse IgG2a isotype mAb, (f) 1A9 mAb, (g) 2D3 mAb, (h) 2D8 mAb, (i) 2E2 mAb, (j) 2E6 mAb, (k) 3C12 mAb, or (l) 3H4 mAb. Monocyte adhesion was then quantified and compared based on the (m) mean fluorescence intensity of sample wells measured by spectrophotometry and the (n) number of adhered monocytes per mm2 calculated from cell counts of 3 random fields of view observed at 20× magnification (0.5 mm2). Graph M shows the mean +/- SD of 4 independent experiments with 3 sample replicates each (total N = 12) and Graph N shows the mean of 2 independent experiments with 3 sample replicates each and 2 independent experiments with 2 sample replicates each (N = 10) analyzed by ANOVA and post-hoc Tukey testing. Asterisk labels indicate statistical significance of the non-treated and mAb-treated groups compared to the LPS-stimulated group unless otherwise specified. ns *p* > 0.05, ^*^p < 0.05, ^**^p < 0.01, ^***^p < 0.001, ^****^p < 0.0001.

The static adhesion assay was then employed to assess the potential therapeutic effects of the anti-VCAM-1 mAb panel generated from phage display biopanning by Perera *et al*. [11] Using the commercial anti-VCAM-1429 control mAb from the preliminary assay as a reference, we evaluated the inhibitory activity of each of the seven candidates on adhesive monocyte-endothelial interactions. For quantitative analysis, the signal intensity of each well was measured by fluorescent spectrophotometry (Fig. 2m), and the number of adherent cells per unit area (Fig. 2n) was calculated from fluorescent microscopy images (Fig. 2a–l). As expected based on the scFv results [11], several of the mAb candidates effectively reduced (*P* < .05) monocyte adhesion compared to the LPS-stimulated control group (Fig. 2b) (Supplementary Table S1) [11]. Based on the spectrophotometry data (Fig. 2m), three mAbs from the candidate panel demonstrated levels of efficacy (Supplementary Table S2) that were more statistically similar to the commercial anti-VCAM-1 antibody: 2E2 (*P* > .9999), 3C12 (*P* = .6409), and 3H4 (*P* = .1604). Notably, pre-incubating the inflamed cell monolayers with a mouse IgG_2a_ isotype negative control antibody (Fig. 2d) did not significantly reduce (*P* > .05) monocyte adhesion compared to the LPS-treated group, supporting the conclusion that the blockade in adhesion is a VCAM-1-mediated effect. Although we cannot rule out an impact of Fc receptors binding the anti-VCAM-1 antibodies on our study, our data clearly shows that our mouse anti-mVCAM-1 antibody can significantly adhesion of murine monocytes to murine endothelial cells. A similar result was obtained by Park *et al.* [10] with their fully human H6 and 7H anti-hVCAM-1 antibodies on HUVECs and U937 cells.

### VCAM-1 antibodies decreased leukocyte rolling and adhesion on endothelial microvessels under flow conditions

**Figure 3:**
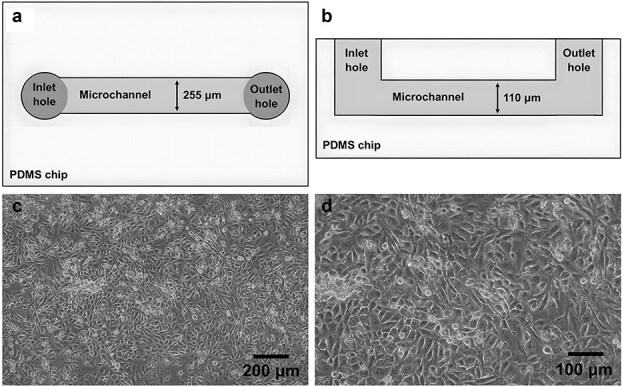
The microfluidics-based endothelial microvessel model used for the flow adhesion assay. (a-b) Schematic diagram of the single-channel microfluidics chip illustrated from the (a) top and (b) side view. The channel dimensions are 255 µm × 110 µm (W × H). (C-D) Brightfield microscopy images were taken at (c) 10× and (d) 20× magnification to confirm the successful formation of an endothelial monolayer over the microchannel surface. All representative images have been taken from a series of 3 independent experiments (N = 3).

**Figure 4:**
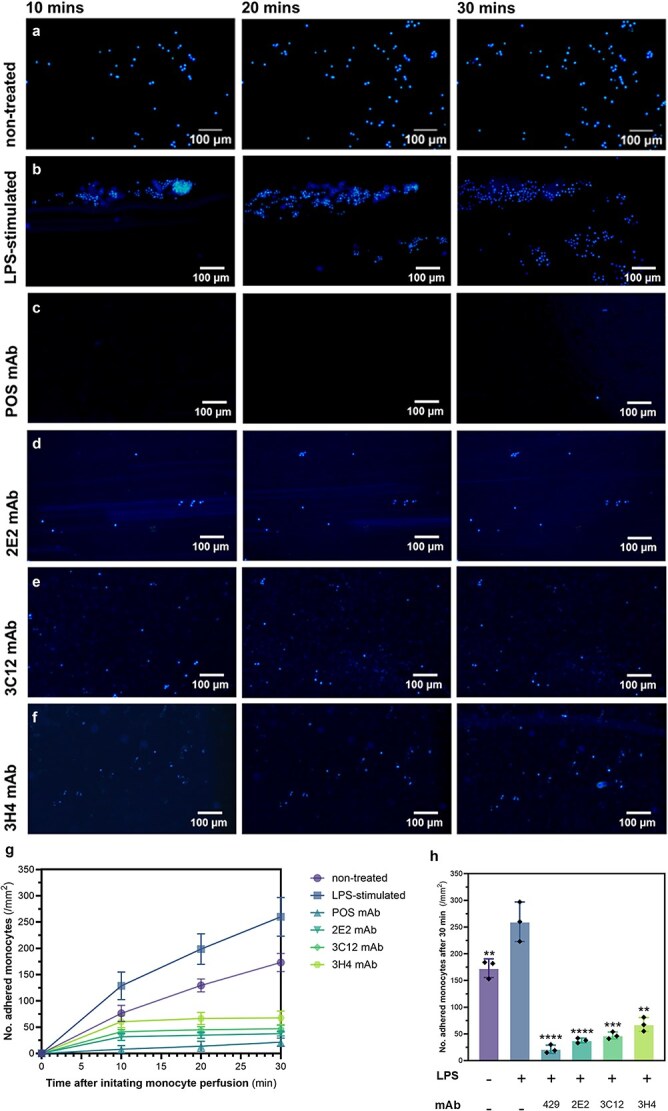
Testing the ability of novel anti-VCAM-1 mAb candidates to inhibit monocyte adhesion under flow using the endothelium-on-the-chip model. Hoechst-labelled RAW264.7s were perfused through LPS-stimulated and anti-VCAM-1 mAb-treated SVEC4-10 microvessels seeded within single-channel microfluidics chips. (a-f) Representative fluorescent images of monocyte adhesion to endothelial microvessels taken at 10, 20, and 30 min after initiating monocyte perfusion following (a) no treatment, (b) LPS stimulation, or LPS stimulation followed by treatment with (c) positive control anti-VCAM-1 429 mAb, (d) 2E2 mAb, (e) 3C12 mAb, and (f) 3H4 mAb. (g) Cell adhesion was quantified at each time point by automatically counting labelled monocytes in 4 random fields of view observed at 20× magnification (0.5 mm2). (h) Group comparison of control and treatment groups at the 30-min time point. Graphs show the mean +/- SD of 3 independent experiments (N = 3) experiments with one replica per experiment analyzed by ANOVA and post-hoc Tukey testing. Asterisk labels indicate statistical significance of the non-treated and mAb-treated groups compared to the LPS-stimulated group unless otherwise specified. ns *p* > 0.05, ^*^p < 0.05, ^**^p < 0.01, ^***^p < 0.001, ^****^p < 0.0001.

Following the static adhesion assay, the three most effective antibodies– 2E2, 3C12, and 3H4 –were tested on a microfluidics-based vascular platform that observed leukocyte rolling and arrest under physiological flow conditions. This model consisted of a 3D endothelial microvessel enclosed within a PDMS microfluidic chip template and subjected to constant peristaltic flow. The channel design and dimensions of the microfluidic chip (Fig. 3a, b) had been determined prior to this study in previous experiments by Akther *et al.* [19] modelling the vascular environment during inflammatory disease. While the SVEC4–10 and RAW264.7 cell lines employed in this model are murine in origin, the peristaltic flow rate (31 μl/min) was intentionally chosen to mimic the physiological arterial shear rate (1000 s^−1^) observed in the human vasculature [19]. Brightfield microscopy images (Fig. 4c, d) confirmed the successful formation of a confluent endothelial monolayer on the microchannel surface that could be maintained under constant exposure to flow for several days. Using brightfield and fluorescent microscopy techniques, the microfluidic model allows for the real-time visualization and quantification of adhesive cell-to-cell interactions between rolling monocytes and endothelial microvessels under physiological flow conditions. Therefore, this device could be utilized as an *in vitro* platform for modelling and predicting the therapeutic efficacy of our anti-VCAM-1 mAb candidates on monocyte-endothelial interactions in the human vascular microenvironment.

This microfluidic model of vascular inflammation was then utilized to determine whether the three selected test antibodies could inhibit leukocyte rolling and adhesion under physiological flow conditions. In brief, endothelial microvessels were pre-incubated with LPS for inflammatory stimulation, administered with test antibody, and then perfused with fluorescently labelled monocytes for the flow adhesion assay. Cell attachment was visualized by fluorescent microscopy images (Fig. 4a–f) taken at 10-min intervals after initiating monocyte perfusion and then subsequently quantified from automatic cell counting. Figure 4g demonstrates that LPS stimulation (Fig. 4b) significantly increased (*P* < .05) monocyte adhesion to the endothelial microvessel at all time points compared to the non-treated group (Fig. 4a). Furthermore, VCAM-1 immunoblockade treatment with the commercial anti-VCAM-1429 mAb (Fig. 4c) was able to significantly reduce (*P* < .01) monocyte adhesion to stimulated endothelia, much like in the static adhesion assay (Fig. 4).

For both the non-treated and LPS-stimulated control groups, cell attachment increased consistently between each of the 10-min time points (Fig. 4g). Like in the static adhesion assay, the amount of cell attachment under flow was significantly higher (*P* < .05) with LPS treatment at all the measured time points compared to the non-treated group (Fig. 4a, b, g, h). As expected, all the antibody-treated groups were able to maintain stable levels of minimal cell adhesion after the initial amount of monocyte attachment recorded at the 10-min time point. Of the novel mAb candidates, 2E2 was the most effective in blocking monocyte-endothelial interactions under flow, decreasing the number of adhered monocytes at 30-min by 85.5% compared to the LPS-stimulated group (Fig. 4b, d, g, h). To note, unlike in the static adhesion assay (Fig. 2), the antibody-treated groups decreased the number of adhered monocytes under flow to a level significantly below (*P* < .05) that observed for the non-treated control group. This observation suggests that our microfluidics-based assay model involves background levels of adhesion that are not attributed to inflammatory stimulation by LPS. Endothelial expression of VCAM-1 without chemical stimulation has been reported in the literature, in which shear stress upregulates surface VCAM-1 and increases monocyte adhesion *via* inducible cell signalling pathways [27, 28], which could explain our results. Our findings are consistent with previous studies demonstrating the capacity of anti-VCAM-1 mAbs to decrease monocyte rolling and adhesion on atherosclerotic carotid arteries *ex vivo* [29, 30].

### VCAM-1 antibodies reduced leukocyte transmigration into 3D cell-hydrogel constructs under static conditions

**Figure 5:**
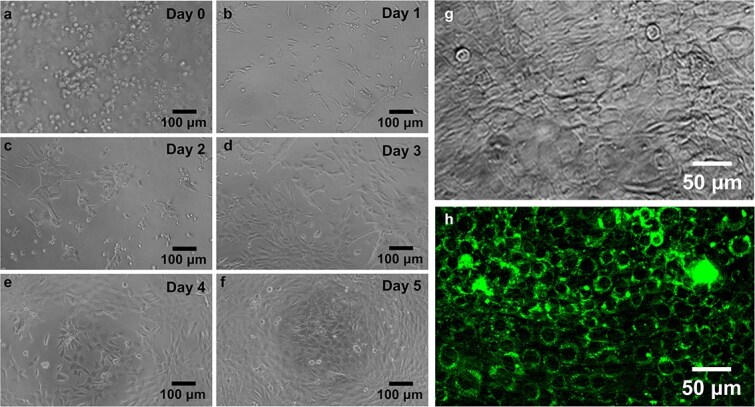
Establishing a confluent endothelial monolayer on top of the cell-hydrogel constructs. (a-f) Brightfield microscopy was taken at 20× magnification each day after seeding and before refreshing media to monitor endothelial cell growth and spreading on the hydrogel surface. VE-cadherin immunostaining was performed once cells reached confluency to visually confirm endothelial cell contact integrity. Representative (g) brightfield and (h) fluorescence microscopy images at 40× magnification show homogenous distribution of fluorescently labelled VE-cadherin, indicating that adherens junctions had been well-established between confluent endothelial cells. Shown are representative images of 4 independent experiments, with one replica per experiment (N = 4).

To generate a comprehensive picture of monocyte recruitment during atherosclerotic lesion formation, it is necessary to evaluate the effects of drug candidates not only on cell adhesion, but also on cell migration. For this reason, we tested the three selected novel anti-VCAM-1 mAbs—2E2, 3C12, and 3H4—on a hydrogel-based cell model used previously to model monocyte recruitment and foam cell formation *in vitro* [21]. The cell-hydrogel construct model employed for our purpose comprised an endothelial/smooth muscle cell co-culture involving an SVEC4–10 monolayer seeded atop a MOVAS-laden collagen gel matrix. To confirm the successful formation of a confluent endothelial monolayer atop the hydrogel surface, we monitored daily cell proliferation using brightfield microscopy (Fig. 5a–f). VE-cadherin immunostaining visually confirmed endothelial contact integrity, with the homogenous distribution of fluorescence indicating adherens junctions had been established between endothelial cells at confluency (Fig. 5g, h). Therefore, this hydrogel-based model could be used to evaluate the effects of our novel mAb candidates on endothelial barrier function and vascular permeability by quantifying monocyte transmigration into the gel matrix.

**Figure 6:**
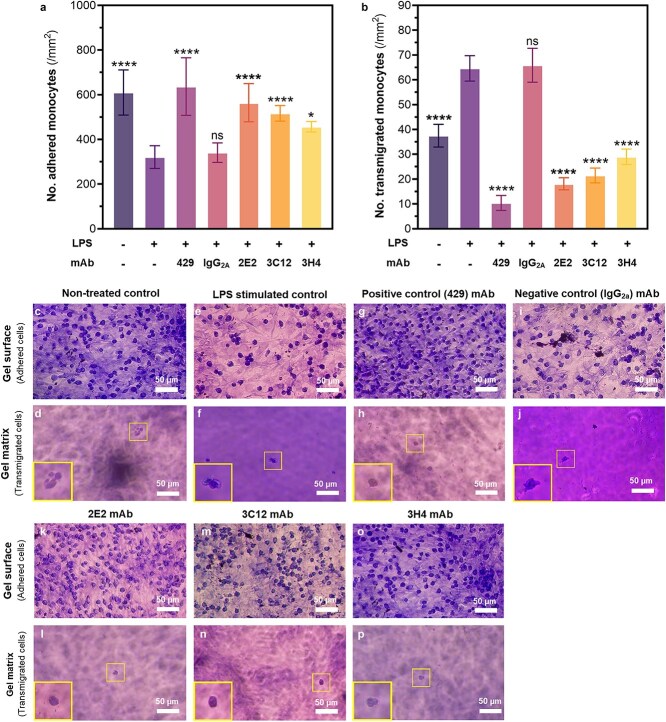
Visualizing monocyte adhesion and transmigration within the cell-hydrogel constructs using Giemsa staining and brightfield microscopy. Endothelialized cell-hydrogel constructs were incubated with monocytes to observe cell attachment and migration, then fixed and stained for visualization. Preliminary cell counts of (a) cell adhesion and (b) transmigration per unit area (/mm2) of hydrogel were performed for group comparison. Representative brightfield microscopy images were taken at 20× magnification to visualize adhered cell on the endothelialized hydrogel surface and within the gel matrix (c-d) non-treated, (e-f) LPS-stimulated, (g-h) anti-VCAM-1 429 mAb-treated, (i-j) negative isotype control-treated, (k-l) 2E2 mAb-treated, (m-n) 3C12 mAb-treated, and (o-p) 3H4 mAb-treated cell-hydrogel models. Stained monocytes were counted from 4 random fields of view observed at 20× magnification (0.5 mm2). Yellow boxes depict close-up images of individual monocytes at 40× magnification. Graphs show the mean +/- SD of 4 independent experiments with 2 technical replicates (total N = 8) analyzed by ANOVA and post-hoc Tukey testing. Asterisk labels indicate statistical significance of the non-treated and mAb-treated groups compared to the LPS-stimulated group unless otherwise specified. ns *p* > 0.05, ^*^p < 0.05, ^**^p < 0.01, ^***^p < 0.001, ^****^p < 0.0001.

Using this hydrogel-based 3D cell culture model, we investigated the ability of our novel anti-VCAM-1 mAb candidates to inhibit monocyte-endothelial adhesion and transendothelial migration *in vitro*. To do this, LPS-stimulated cell-hydrogel constructs were pre-treated with 20 μg/ml anti-VCAM-1 mAbs for 1 h and then incubated with monocyte cell suspensions for 1 h before replenishing the media and incubating for a further 24 h. As demonstrated in Fig. 6, we were able to visually differentiate adhered monocytes upon the endothelialized hydrogel surface (Fig. 6c–e, i–k) from transmigrated monocytes deeper within the hydrogel matrix (Fig. 6f–h, l–n) using a combination of brightfield microscopy and Giemsa staining. After imaging, preliminary cell counts were conducted to observe how LPS stimulation and anti-VCAM-1 mAb treatment affected the proportion of monocytes that had adhered and transmigrated per unit area of the gel constructs. Contrary to the previous adhesion models, the number of adhered monocytes on the gel surface was significantly lower (*P* < .05) for the LPS-stimulated group compared to all other test groups (Fig. 6a). However, the number of transmigrated monocytes in LPS-stimulated group was significantly higher than the non-treated and mAb-treated groups (Fig. 6b).

Importantly, all antibody-treated groups exhibited monocyte adhesion levels comparable to the baseline (unstimulated) condition, indicating that the anti-VCAM-1 antibodies effectively blocked VCAM-1 availability in LPS-stimulated cells. Moreover, these antibodies significantly reduced monocyte transmigration compared to both LPS-stimulated and unstimulated conditions, further highlighting their excellent performance. The low number of adhered monocytes in the LPS-stimulated group can be attributed to their rapid transmigration into the hydrogel construct. When examining the two graphs together, the data does indeed suggest that the LPS-stimulated group exhibited a higher degree of transmigration compared to the other test groups, resulting in a significantly lower proportion of monocytes remaining adhered to the endothelial surface. Overall, the trend observed with the antibody-treated groups would suggest that VCAM-1 treatment effectively inhibited both the monocyte adhesion and transmigration steps of the recruitment cascade.

**Figure 7:**
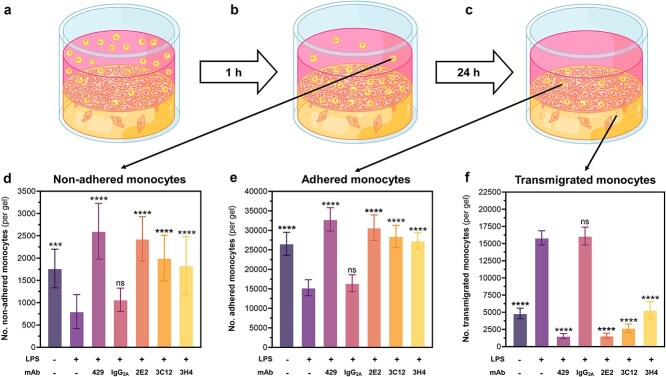
Comparing monocyte adhesion and transmigration of monocytes in LPS-stimulated endothelialized 3D collagen constructs with novel anti-VCAM-1 mAb treatments. As described in the experimental section, (a) monocytes were added onto the cell-hydrogel constructs and left for 1 h to allow adhesion. (b) After this time point, non-adhered monocytes in the supernatant were collected and the constructs were left to incubate for a further 24 h. (c) After this incubation period, adhered and transmigrated monocytes were separated and quantified by subsequent trypsinization and collagen gel digestion. (d) Non-adhered cells were collected by rinsing cell-hydrogel constructs with DPBS, pooling the supernatants, and counting cells using the haemocytometer. (e) Adhered cells were quantified by detaching the endothelial monolayer with trypsin, selectively Giemsa staining monocytes, and counting using a Giemsa smear with brightfield microscope. (f) Transmigrated cells were collected by digesting the cell-hydrogel constructs in collagenase, selectively staining monocytes with Giemsa, and counting all stained cells within a Giemsa smear. Graphs show the mean +/- SD of 4 independent experiments with 3 sample replicates each (total N = 12) analyzed by ANOVA and post-hoc Tukey testing. Asterisk labels indicate statistical significance of the non-treated and mAb-treated groups compared to the LPS-stimulated group unless otherwise specified. ns *p* > 0.05, ^*^p < 0.05, ^**^p < 0.01, ^***^p < 0.001, ^****^p < 0.0001.

As the results obtained from microscopy analysis only accounted for the number of cells per unit area of the 3D cell constructs, we performed a second quantification procedure to calculate the number of non-adhered, adhered, and transmigrated monocytes throughout the entire matrix volume. This method allowed us to account for possible heterogeneity of cell distribution throughout the hydrogel constructs and common visual interpretation biases from microscopy imaging [31]. After initiating the cell transmigration assay as described above, monocytes were collected and counted in the three stages of recruitment– non-adhesion, adhesion, and transmigration –using a conventional Giemsa smear method. Firstly, non-adhered monocytes were retrieved from the media supernatant left after incubating cell-hydrogel constructs with monocyte suspension for 1 h (Fig. 7b). Secondly, adhered monocytes were obtained after an additional 24 h incubation period by trypsinizing and resuspending the endothelial monolayer seeded atop the hydrogel surface (Fig. 7c). Finally, the transmigrated monocytes inside the gel matrix were recovered by collagenase digestion of the remaining hydrogel constructs (Fig. 7c). The selective nature of Giemsa staining for leukocytes allowed these cells to be easily distinguished from the other endothelial and smooth muscle cells in the complete co-culture [24].

After the first monocyte treatment period (Fig. 7b), the static transmigration assay could be used to indirectly determine the effects of VCAM-1 impact on monocyte adhesion by quantifying the number of non-adhered monocytes. As expected, LPS stimulation increased initial monocyte adhesion to endothelial monolayers (Fig. 7d), as demonstrated by a significantly lower number (*P* < .01) of non-adhered cells free floating in the supernatant compared to the non-treated endothelium. Furthermore, anti-VCAM-1 mAb treatment effectively reversed this effect, with all four mAbs exhibiting significantly higher (*P* < .05) numbers of non-adhered cells compared to the LPS-stimulated group. Then, after the second monocyte incubation period (Fig. 7c), the proportion of adhered and transmigrated monocytes was quantified. Much like in the preliminary cell count of monocyte adhesion (Fig. 7a) calculated from the brightfield microscopy method, Fig. 7e shows that the number of monocytes adhering to the endothelium after 24 h was significantly lower (*P* < .05) for the LPS-stimulated group compared to the non-treated and antibody treated groups. Even more, the number of transmigrated monocytes was significantly higher (*P* < .01) for the LPS-stimulated group compared to the other test groups (Fig. 7f). Taken together, the results show that the positive control mAb and the three mAb candidates were successful in blocking monocyte transmigration—with the number of transmigrated monocytes decreasing by 89.2% after treatment with the 429 mAb, 89.5% with 2E2 mAb, 84.6% with 3C12 mAb, and 82.3% with 3H4 mAb (Fig. 7e, f). Importantly, the cumulative results of the non-adhered, adhered, and transmigrated cell counts show that our VCAM-1 mAbs effectively inhibited monocyte recruitment at two separate steps of the cascade– cell adhesion and cell transmigration.

## Discussion

The pathophysiological role of VCAM-1 in atherosclerosis has been well documented [32]. This association has seen VCAM-1 utilized as a targeting moiety in several antibody- and peptide-conjugated drug delivery systems for site-specific visualization and treatment of atherosclerotic lesions [9]. However, research into the therapeutic mechanisms of anti-VCAM-1 antibodies outside of these targeting abilities remains strikingly limited [8]. In addition to mediating leukocyte-endothelial adhesion and trafficking, VCAM-1 regulates endothelial signalling pathways related to oxidative stress and vascular permeability [33]. Considering this, researchers may be able to precisely modulate the anti-inflammatory activities of VCAM-1-targeted therapeutics by designing peptides and antibody antagonists that target specific domains of the VCAM-1 protein. In previous experiments, Perera *et al.* [11] generated a panel of novel scFvs against VCAM-1 using phage display biopanning technology, which appear to bind different domains of VCAM-1, and which were subsequently reformatted into mAbs to be tested in this study. Through a series of competitive binding (Fig. 2 and Fig. 4) and migration (Fig. 6 and Fig. 7) assays representing vascular inflammation *in vitro*, we were able to evaluate the therapeutic potential of our mAb candidates for atherosclerosis. The initial static adhesion assay depicted in Fig. 2 demonstrated that all candidates of the mAb panel were able to significantly reduce (*P* < .05) monocyte attachment to LPS-stimulated endothelial monolayers—with 2D8, 3H4, 3C12, and 2E2 mAbs being the most effective (listed in order of least to greatest impact on binding). Our results using mouse anti-VCAM-1 antibodies to block murine monocyte adhesion to murine endothelial are similar to Park *et al.*’s [10] human anti-VCAM-1 mAbs in a human cell adhesion assay. Together, these results demonstrate that binding to monocytes’ Fc receptors does not prevent the antibodies’ ability to significantly reduce cell adhesion, a result directly relevant to future clinical applications.

Several endothelial ligands—each exhibiting overlapping and complementary involvement in cell recruitment—could be considered drug targets for atherosclerosis therapy. The inducible nature of VCAM-1 means that it is selectively upregulated at sites of vascular dysfunction, marking it as an especially attractive target for precise and localized therapeutic activity [34]. As selectively inhibiting one specific vascular receptor leaves the opportunity for compensation by other related ligands, choosing a drug target that is functionally involved in all the stages of the transendothelial migration cascade is essential. The interaction between VCAM-1 on activated vascular endothelial cells and α_4_ integrin on circulating monocytes is critical in mediating the transition from weak leukocyte rolling and adhesion to firm arrest and transmigration [8, 35]. The standard microplate (Fig. 2) and microfluidics-based (Fig. 4) adhesion assays employed in this study demonstrated that our mAb candidates were able to inhibit both weak monocyte-endothelial adhesions in a static environment and stronger adhesive interactions under flow. Furthermore, we were able to show that the mAbs were able to inhibit both adhesion and transmigration by employing the 3D hydrogel model (Fig. 6 and Fig. 7) despite the possibility of compensation by other endothelial receptors. Dose-response studies were not conducted as part of this study, though future investigations of multiple treatment concentrations would greatly improve comparisons on the potential therapeutic efficacy of the mAb candidate panel.

VCAM-1 is predominantly expressed in humans as a seven-domain protein in which the first, second, and third domains are highly homologous to the fourth, fifth, and sixth domains, respectively [36]. Domains 1 and 4 have both been implicated in monocyte attachment due to the presence of integrin-binding isoleucine-aspartate-serine-proline-leucine (IDSPL) amino acid sequence motifs recognized by complementary α_4_β_1_ and α_4_β_7_ integrins [37]. Naturally, these two domains are considered target epitopes for anti-VCAM-1 antibodies to block pathological cell recruitment during inflammatory disease. However, a few studies have developed antibodies to domains other than 1 and 4, suggesting the possibility of interfering with VCAM-1 function via these non-α_4_β_1_ and α_4_β_7_ integrin binding domains [10, 12, 38]. The positive control 429 antibody used in this study inhibits domains 1 and 2 [11]. Of the four most effective mAb candidates tested in this study, 2D8 and 2E2 have both previously demonstrated binding to domain 3 of murine VCAM-1 [11], whereas 3H4 binds to domain 2 [11], and 3C12 binds to domain 1 (data not shown in Perera *et al.* [11]). The ability of 3C12 to inhibit monocyte recruitment can likely be attributed to the competitive blockade of the α_4_ integrin-binding sequence in domain 1 [37]. While the possible mechanisms of the other three mAbs have not been clearly linked to blocking the specific functions of domain 2 or 3, steric hindrance is a likely explanation for their adhesion-blocking effects. The presence of domain 2 has been previously identified as a structural requirement for the binding function of domain 1, whereas the role of domain 3 in VCAM-1 function is yet to be elucidated [39].

Considering the novelty of selectively targeting domain 2 or 3 of VCAM-1 as a strategy for directly combating monocyte recruitment, the tested mAbs represent an intriguing platform for site-specific atherosclerosis nanotherapeutics. The H6 and 7H mAbs previously tested with *in vitro* and *in vivo* models of atherosclerosis by Park *et al.* [10] bound within domains 1 or 2 of VCAM-1. The data presented in our study, however, shows that an anti-VCAM-1 mAb specific to domain 2 and not domain 1– 3H4 mAb –was able to reduce monocyte adhesion (Fig. 2 and Fig. 4) and transmigration (Fig. 6 and Fig. 7) using *in vitro* models of early atherosclerosis. There are also instances in the literature of mAbs against domain 3 of VCAM-1– which is the domain targeted by our 2E2 mAb –demonstrating inhibitory activity against inflammation-induced monocyte recruitment [40–42]. A possible explanation for the effectiveness of the 3H4 and 2E2 mAbs is that the domains they target may possess functional involvement in endothelial signalling pathways necessary for monocyte adhesion and migration. However, this hypothesis would warrant further investigation to determine the effects of our antibody candidates on signal transduction and protein expression. For example, the VCAM-1 protein activates several pathways involved in the clathrin-dependent internalization of VE-cadherin at endothelial cell-to-cell junctions, resulting in increased paracellular permeability during vascular inflammation [8, 33, 43]. The ability of our antibodies to inhibit monocyte transmigration *in vitro* could be interpreted as a preliminary indicator of interference with this pathway, though further study would be necessary to verify this. Of course, steric hindrance is another possibility for the efficacy of the anti-VCAM-1 mAbs, in which the antibodies physically block interactions between VCAM-1 and their complementary ligands. With this in mind, the mechanistic implications and potential synergy of combining treatment with mAbs that target different domains of the VCAM-1 protein is an interesting avenue for future studies with these antibody candidates.

As an important note, VCAM-1 is one of several types of adhesive surface receptors expressed on the activated endothelium during vascular inflammation – including E-selectin, P-selectin, and intracellular adhesion molecule (ICAM). While selectins mediate low-affinity interactions involved in monocyte rolling on the endothelium, VCAM-1 can facilitate firmer, more stable, cell attachments required for transendothelial migration due to its high-affinity binding to α_4_β_1_ integrin on activated monocytes [44]. The ICAM-1 receptor, however, demonstrates high affinity binding to β_2_ integrins on circulating monocytes and can support firm cell attachment and transmigration in a manner similar to that of VCAM-1 [27]. It is therefore important to acknowledge that antibody suppression of VCAM-1 is theoretically unable to completely ablate monocyte transmigration due to compensation by ICAM-1 and, though to a lesser extent, E- and P-selectin. The effect is this receptor compensation is evident from the residual levels of monocyte attachment and transmigration after anti-VCAM-1 mAb treatment observed across all assays employed in this study. Nonetheless, VCAM-1 blockade with therapeutic antibodies can still be considered a viable strategy for inhibiting monocyte recruitment associated with vascular disease progression, especially because VCAM-1 expression is present in 82% of atherosclerotic lesions compared to only 46% for ICAM-1 [45].

The microfluidic and 3D *in vitro* models employed in this study represent novel and sophisticated pre-clinical testing platforms compared to conventional cell culture assays due to their abilities to replicate certain physiological parameters of the human vascular system. For the microfluidic model, the peristaltic flow rate (31 μl/min) perfused through the device had been calculated to match the physiological shear stress (1000 s^−1^) observed in human arteries [19]. Then for the 3D model, the composition of the hydrogel matrix incorporated collagen and smooth muscle cells to simulate the extracellular environment of the subendothelial space within human blood vessels [21]. However, it is also important to acknowledge the limitations of using murine cell lines for modelling human disease processes, especially in terms of clinical translation. In the context of this study, our findings still hold physiological relevance due to the structural and functional similarities between the human and mouse variants of the VCAM-1 protein. All known human and murine isoforms of VCAM-1 contain specific binding sites for the α_4_β_1_ integrin ligand, though with varying degrees of binding affinity [46]. Therefore, the anti-VCAM-1 mAbs in our study could reasonably be predicted to inhibit VCAM-1 activity in human tissues based on the effects on cell adhesion we observed using murine endothelial cells. Nonetheless, it would improve the clinical relevance of these *in vitro* models to modify the designs to incorporate human cell lines as a future research direction.

In conclusion, this study demonstrates the therapeutic potential of a novel panel of anti-VCAM-1 mAbs for treating atherosclerosis by employing a series of *in vitro* models mimicking vascular inflammation. Our results show that all seven antibody candidates were able to effectively inhibit monocyte adhesion under static conditions. Furthermore, the three most effective mAbs– 2E2, 3C12, and 3H4 –were able to inhibit cell adhesion in a flow-based microfluidic model and cell transmigration in a 3D hydrogel construct model. The novelty of these antibody candidates—particularly regarding their binding activity to VCAM-1 domains other than domains 1 and 4—highlights the importance of antibody discovery technology for discovering and identifying new molecular mechanisms for combatting multifactorial disease pathologies such as atherosclerosis.

## Supplementary Material

Pickett_et_al_Supporting_Information_R1_Clean_tbaf025

## Data Availability

Data will be made available upon request.
