# Supplementary material for: Assessing the therapeutic potential of a panel of novel VCAM-1 antibodies using microfluidic and three-dimensional in vitro models of vascular inflammation
Source: Antib Ther. 2025 Nov 4;8(4):350–63. doi: 10.1093/abt/tbaf025 (PMC12683022; doi:10.1093/abt/tbaf025)
Supplement: Pickett_et_al_Supporting_Information_R1_Clean_tbaf025 [file pickett_et_al_supporting_information_r1_clean_tbaf025.docx]

**Supporting Information for**

**Assessing the therapeutic potential of a panel of novel VCAM-1 antibodies using microfluidic and three-dimensional *in vitro* models of vascular inflammation**

**Materials & Method**

## **Materials**

All chemicals were reagent-grade and did not require further purification unless specified otherwise. SYLGARD^TM^ 184 Silicone Elastomer Kit (1317318) was purchased from Dow Corning. Cultrex^®^ 3-D Culture Matrix Rat Collagen I (3447-020-01) was purchased from In Vitro Technologies. Collagen solution from bovine skin (C4243), collagenase D from *Clostridium histolyticum* (11088858001), 3,3-dihexyloxacarbocyanine iodide (DiOC_6_, 318426) Dulbecco’s Modified Eagle Medium (DMEM, D5796), ethanol, Giemsa stain (G4507), isopropyl alcohol, lipopolysaccharide (LPS) from *Escherichia coli* (L2143), methanol, paraformaldehyde (PFA, 16005), and sodium hydroxide were purchased from Sigma-Aldrich. Bovine serum albumin (BSA, A8531), Dulbecco’s phosphate-buffered saline (DPBS, D8537), foetal bovine serum (10099141), Hoechst 33342 staining solution (62249), penicillin-streptomycin (P/S, 15140122), 2.5% (w/v) trypsin (15090046), and TrypLE^TM^ Express enzyme (12604021) were purchased from Thermo Fisher Scientific.

Regarding the control and test antibodies, goat anti-rabbit IgG H&L (Alexa Fluor^®^ 488) mAb (Abcam #ab150077, RRID: AB_2630356) and rabbit anti-mouse recombinant anti-VCAM-1 (EPR5047) mAb (Abcam #ab134047, RRID: AB_2721053) were purchased from Abcam. Rat anti-mouse anti-VCAM-1 (CD106) 429 mAb (Thermo Fisher Scientific #14-1061-82, RRID: AB_467419), rat anti-mouse VE-cadherin (CD144, Alexa Fluor^®^ 488) BV13 mAb (Thermo Fisher Scientific #53-1441-80, RRID: AB_1210528), and mouse IgG isotype control (ThermoFisher Scientific #02-6502, RRID: AB_2532951) were purchased from Thermo Fisher Scientific.

## **Cell culture**

SVEC4-10 (ATCC CRL-2181) endothelial cells, RAW264.7 (ATCC TIB-71) macrophage, and MOVAS (ATCC CRL-2797) smooth muscle cell lines were obtained from the American Type Culture Collection (ATCC). RAW264.7 macrophages were maintained in complete, low-glucose (1 mg/mL) DMEM supplemented with 10% FBS and 1% P/S. SVEC4-10 and MOVAS cells were maintained in complete, low-glucose DMEM supplemented with 10% FBS and 1% P/S. All cell culture vessels were stored at 37 °C in a humidified incubator at 5% CO_2_ atmosphere and passaged following manufacturer guidelines using TrypLE^TM^ Express cell dissociation reagent.

## **Static monocyte adhesion assay on the microplate**

The static monocyte-endothelial cell adhesion assay we employed for competitive binding inhibition studies of the antibody panel was optimized based on a previous adhesion assay protocol described by Park *et al*.^[10]^ SVEC cells (passages 8-10) were plated on a 96-well microtitre plate at a density of 2.0 × 10^4^ cells per well and cultured in DMEM for 24 h at 37 °C. All subsequent treatment steps were performed by introducing 100 µL reagent solution to each well after rinsing with DPBS unless specified otherwise. The seeded SVEC cells were stimulated with 100 ng/mL LPS for 24 h at 37 °C and then pre-treated with 20 µg/mL anti-VCAM-1 mAbs (positive control 429 mAb, IgG isotype negative control mAb, and anti-VCAM-1 1A9, 2D3, 2D8, 2E2, 2E6, 3C12, 3H4 IgG_2a_ clones) for 1 h at 37 °C. Concurrently, RAW macrophages (passages 15-17) were fluorescently labelled by incubating with 50 ng/mL DiOC_6_-supplemented DMEM and then centrifuge-washed (700 × *g*, 5 min, 20 °C) to remove excess stain. The DiOC_6_-labelled macrophages were added to the plated cell monolayers at a density of 1.0 × 10^4^ cells per well and incubated in the dark at 37 °C for 10 min to allow cell attachment. After incubating, all wells were washed twice with DPBS to remove unbound macrophages and fixed with 4% (w/v) PFA at room temperature for 20 min.

## **Fluorescence spectrophotometry and microscopy of static binding assay**

Fluorescence analysis of the static monocyte adhesion assay was performed using two separate quantitative methods: plate reader spectrophotometry and fluorescence microscopy. The cumulative fluorescence of the adherent DiOC_6_-labelled macrophages in each well was measured using a BMG LABTECH CLARIOstar^®^ microplate reader with excitation set to 480 nm and emission set to 520 nm. Samples were blank-corrected against 100 µL DPBS and normalized to the non-antibody-treated, LPS-stimulated control wells for group comparison. Fluorescence microscopy images were captured using the OLYMPUS CKX53 fluorescence microscope with a Cool LED pE-300 light source, OLYMPUS-DP74 camera, and Olympus cellSens^TM^ imaging software. Cell adhesion was quantified from fluorescent microscopy images using Fiji (Version v1.54k for Windows, University of Wisconsin, United States) image processing software.^[14]^ Labelled monocytes within 3 randomly selected fields at 20× magnification (0.5mm^2^/field) were automatically counted within each well, and then the mean number of adherent cells per mm^2^ was calculated.

## **Microfluidics chip fabrication**

Microfluidics chips were fabricated according to standard photolithography and polydimethylsiloxane (PDMS) soft lithography techniques.^[18]^ PDMS pre-polymer was mixed with SYLGARD^TM^ 184 Silicone Elastomer at a 10:1 mass ratio to create a solid elastomer with optimum biocompatibility for cell culture. The PDMS mixture was degassed in a desiccator for 5-10 min, poured onto a silicon master mould corresponding to the appropriate channel design, and then cured at 75 °C for 2 h to allow the PDMS to solidify. Once the PDMS slabs were separated from the master mould, the edges were cut, and then inlet and outlet holes were punched with a 1 mm-diameter needle. The slabs were then washed with 70% (v/v) isopropyl alcohol and distilled water and then dried at 75 °C for 5 min. Finally, the PDMS chip was tightly sealed to a clean glass slide by treating it with oxygen plasma for 1.5 min using a Harrick Plasma PDC-32G Plasma Cleaner. For this study, the design of the microfluidic device consisted of a single rectangular microchannel chamber (255 µm wide and 100 µm high) with circular inlet and outlet ports (100 µm in diameter).

## **Microvessel formation in the microfluidics device**

Endothelial cells were seeded within the microfluidic devices using a protocol previously reported by Akther *et al.*^[19]^ After chip fabrication and bonding, the microfluidic device was sterilized by exposing it to ultraviolet light for 1 h, perfusing the microchannel with 70% (v/v) ethanol for 10 min, and then washing twice with DPBS. The inner surface of the microchannel was then functionalized with 0.4 mg/mL bovine skin collagen solution to counter the inherent hydrophobicity of PDMS for optimal endothelial cell adhesion to the PDMS substrate.^[20]^ For cell seeding, a 1.5 × 10^7^ cells/mL suspension of SVEC cells (passage <10) was injected through the inlet port of the microchannel by manual pipetting. The chip was then incubated at physiological temperature with orbital shaking (200 rpm) for 1 h to expose the system to oscillatory flow during the period of initial cell-substrate attachment. Afterwards, the device was incubated at 37 °C and 5% CO_2_ for an additional 4 h, flipping the chip at the halfway time point to support the formation of a complete 3D microvessel covering all faces of the rectangular microchannel.^[19]^

Before commencing with the flow-based cell adhesion assay, the seeded SVEC cells were cultured in the microchannel under controlled flow conditions to allow the formation of a uniform endothelial microvessel. The microfluidic device was attached to a peristaltic pump (NE-9000 PeriPumpONE New Era Pump Systems) via the inlet and outlet ports of the microchannel to subject the microvessel to continuous peristaltic flow. All components of the peristaltic circuit were connected using silicon tubing (1.5 mm diameter), which had been sterilized beforehand with 70% (v/v) ethanol for 30 min and ultraviolet light for 1 h. Once attached to the pump circuit, the microfluidic device was maintained at 37 °C and 5% CO_2_ until required for microscopy. For the first 12 h of incubation, the pump was initially set to a low flow rate (12 µL/min) to avoid disrupting cell-substrate attachment and proliferation while still exposing the cell monolayer to moderate shear stress. The flow rate was then increased to representative blood flow rate (31 µL/min) for a further 24 h to allow sufficient time for the seeded cell monolayer to form a uniform endothelial microvessel. This flow rate was calculated to equate to typical arterial shear rate (1000 s^-1^) based on the dimensions of the microchannel and Newton’s Law of Viscosity.^[21]^ All subsequent treatments introduced into the microfluidic device were perfused at this flow rate unless specified otherwise.

## **Flow-based monocyte adhesion assay on the microfluidic chip**

Once a confluent monolayer covering all faces of the rectangular microchannel had been established, the microvessel was stimulated by perfusing 1 µg/mL LPS-supplemented DMEM through the microfluidic circuit for 8 h. Using the same perfusion technique, devices were subjected to anti-VCAM-1 mAb (429 positive control mAb, and 2E2, 3C12, 3H4 IgG_2a_ clones) pre-treatment for 2 h before commencing with the flow adhesion assay. For this step, RAW macrophages were fluorescently labelled with Hoechst stain (1 µg/mL, 30 min, 30 °C) and injected through the microchannel using a syringe pump (NE-1000 Master Dual Syringe Pump, New Era Pump Systems) to observe real-time monocyte rolling and adhesion under continuous flow. Fluorescence microscopy images were taken at three 10-min intervals after initiating monocyte perfusion into the microchannel. Cell adhesion was quantified from microscopy images using an automatic cell counter in Fiji. Using a similar approach to that described in **Section 2.5**, the number of adherent cells per mm^2^ was calculated by automatically counting Hoechst-labelled monocytes within 3 randomly selected fields on the microchannel surface at 20× magnification (0.5 mm^2^/field).

## **Formation of cell-hydrogel constructs for three-dimensional cell culture**

Cell-hydrogel constructs were prepared from Cultrex® Rat Collagen I and MOVAS smooth muscle cells (passage <10) according to the manufacturer’s protocol. In summary, 2 mg/mL collagen and 1.0 × 10^5^ cells/mL MOVAS cells were suspended in high-glucose (5 mg/mL) DMEM and neutralized to pH 7 with 1M NaOH (e.g. add 8 µL of 1M NaOH to 500 µL of hydrogel). The collagen and smooth muscle cell components of the resultant hydrogel matrices served to model the extracellular microenvironment of the vascular subendothelial space.^[22]^ For our 3D transmigration experiments, 50 µL gel precursor mixture per well was added to a tissue culture-treated 96-well microtitre plate and incubated (37 °C, 5% CO_2_) for 1 h to allow the gels to set. After gel solidification was confirmed, hydrogel constructs were endothelialized with an SVEC cell monolayer using a protocol previously reported by Akther *et al.*^[23]^ Briefly, 100 µL of 2.0 × 10^5^ cells/mL SVEC suspension in high-glucose DMEM was added to each gel-coated well and cultured for 5 days to allow a confluent endothelial monolayer to form. Media was refreshed every 24 h to maintain the overall viability of the cell-hydrogel constructs and endothelial monolayers.

Endothelial barrier integrity of the confluent monolayers was confirmed using a standard VE-cadherin immunostaining procedure based on previous methods obtained from the literature.^[24, 25]^ Endothelialized cell-hydrogel constructs were prepared as described in Section 2.6.1 to facilitate the formation of a confluent EC monolayer on the hydrogel surface. For all steps of immunostaining, 100 µL of reagent solution was introduced and incubated at RT unless specified otherwise. The cell-hydrogel constructs were fixed with 4% PFA for 1 h, rinsed once with DPBS, blocked with 1% BSA, and then rinsed again with DPBS. The cells were then labelled by incubating with eBioScience^TM^ CD144 mAb (diluted in 1:100 in DPBS) at 4°C O/N. Once immunostaining was completed, the constructs were rinsed 3 times with DPBS before performing brightfield and fluorescent microscopy imaging for qualitative analysis of endothelial barrier integrity.

## **Monocyte transmigration assay under static, non-flow conditions**

Once the endothelial monolayers were grown to confluency upon the hydrogel, the cells were incubated (37 °C, 5% CO_2_) with 100 ng/mL LPS for 24 h to stimulate endothelial activation during vascular disease. Cell-hydrogel constructs were then pre-treated with 20 µg/mL anti-VCAM-1 mAbs (429 positive control, and 2E2, 3C12, 3H4 IgG_2a_ clones) and incubated for 1 h. After rinsing the constructs once with DPBS, 2.0 × 10^4^ RAW macrophages were added to each well and incubated for 1 h to allow enough time for initial monocyte-endothelial attachment while ensuring enough cells remained in the supernatant for meaningful comparison between treatment groups. To collect the non-adhered cells for counting, the gel constructs were rinsed twice with 100 μL of DPBS, then the pooled supernatants and rinse solutions (300 μL total) from each well were aliquoted. The total number of non-adhered cells from each well was calculated using a conventional haemocytometer counting method. The monocyte-treated constructs were then replenished with fresh DMEM and incubated for an additional 24 h to ensure measurable levels of cell migration occurred before commencing with further analysis.

## **Visual assessment of monocyte transmigration by Giemsa stain microscopy**

To qualitatively assess the effects of inflammatory stimulation and antibody pre-treatment on monocyte transmigration across endothelial monolayers, we treated the cell-hydrogel constructs with Giemsa stain and performed brightfield microscopy analysis. Giemsa stain selectively stains leukocytes, allowing macrophages to be effectively differentiated from endothelial and smooth muscle cells in the hydrogel matrix.^[26, 27]^ Before commencing Giemsa staining, hydrogel constructs were fixed with 4% PFA at room temperature for 1 h. All steps were performed by incubating cell-hydrogel constructs with 100 µL reagent solution at room temperature unless specified otherwise. The protocol was as follows: the cells were fixed with 50% (v/v) methanol for 5 min, 78% (v/v) methanol for 15 min, stained with 5% (w/v) Giemsa working solution (diluted in 1:3:16 glycerine-to-methanol-to-water) for 20 min, then washed thoroughly with water to remove excess stain. For brightfield microscopy, gels were removed from the 96-well plate and placed onto a glass coverslip face down so that the endothelial monolayer was in contact with the glass surface. Brightfield focus was initially adjusted so that the endothelial monolayer was in the field of view, then shifted into the hydrogel matrix to count the total number of transmigrated monocytes. As a preliminary cell counting technique, adhered monocytes on the endothelial monolayer and transmigrated monocytes in the gel matrix were quantified per unit area from a randomly selected field of view for each gel using a similar method to that previously employed for the static adhesion assay.

**Quantitative measurement of transmigration by Giemsa smear**

After qualitative microscopy analysis, an adapted Giemsa smear method was employed to quantitatively compare the inhibitory effects of our anti-VCAM-1 mAb candidates on monocyte adhesion and transmigration under static conditions. Cell-hydrogel constructs were fabricated, and the monocyte transmigration assay was performed as described in **Sections 2.10** and **2.11**. Non-transmigrated monocytes on the hydrogel surface were collected by incubating the cell-hydrogel constructs with 100 µL of 2.5% (w/v) trypsin at room temperature and washing thoroughly with an equivalent volume of DPBS until the endothelial monolayer had been entirely detached. The supernatant and rinse solutions were then pooled (200 μL total) for each gel, centrifuged at 300 × *g* for 5 min, and aliquoted for Giemsa smearing and cell counting. Monocytes were selectively stained for counting using a modified Giemsa smear protocol adapted from the literature.^[28]^ Briefly, a 10 µL suspension of the adherent monocytes and endothelial cells detached from the hydrogel substrate after trypsinization was smeared onto a clean glass cover slip. The cover slip was dipped in 100% (v/v) methanol for cell fixation, air-dried for 30 s, and then stained with Giemsa working solution at room temperature for 30 min before rinsing with deionized water. All Giemsa-stained monocytes within the smear were visualized and counted under a brightfield microscope, and the total number of adhered monocytes per gel was calculated.

To collect transmigrated cells encapsulated within the gel matrix, the trypsinized hydrogels were digested in 100 µL of 0.25% (w/v) collagenase D in DPBS and incubated at 37 °C for 10-15 min until disaggregation was observed. As with the rinse solutions, the liquified gels were collected, centrifuged, resuspended, and stored on ice until ready for the Giemsa staining procedure. Transmigrated monocytes were stained for cell counting and analysis using the same Giemsa smear method described above.

**Statistical analysis**

All data was expressed as mean ± standard deviation of the mean (SDM) and annotated with degrees of significance (*p < 0.05, **p < 0.01, ***p < 0.005, ****p < 0.001). The total number of experimental replicates (N), which includes both independent experiments and sample replicates, are indicated in the figure legends for each experiment. The mean differences of monocyte adhesion and transmigration between antibody-treated groups and controls were analysed using one-way analysis of variance (ANOVA) followed by post-hoc Tukey for pairwise comparisons. Values of p < 0.05 were considered statistically significant. All data visualization and statistical analyses were conducted with GraphPad Prism 10 (Version 10.3.1 for Windows, GraphPad Software, San Diego, United States).

**Results**


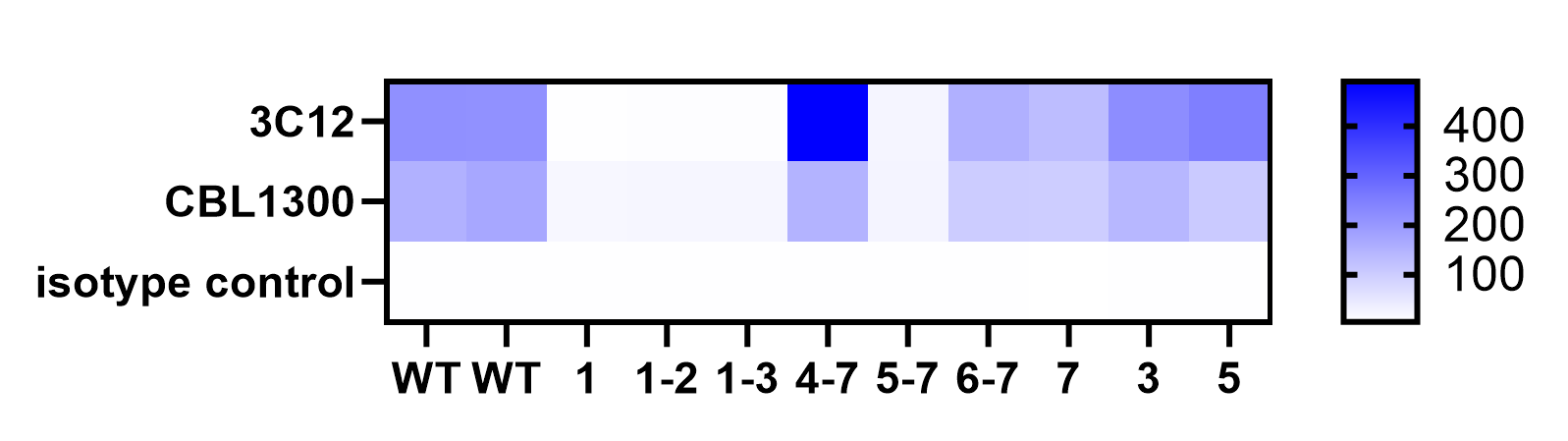


**Supplementary Figure S1:** Clone 3C12 binds Ig-like domain 1 of mVCAM-1. The 3C12 clone was developed by Perera *et al.* and tested simultaneously with the other clones described in that manuscript (please refer to Figure 4, Results, and Methods in Perera *et al.*). Binding of the 3C12 clone to different domains of VCAM-1 was assessed by flow cytometry, using Ig-like domain deletion versions of VCAM-1 C-terminally tagged with GFP and N-terminally tagged with FLAG (to show extracellular expression) transiently expressed in CHO cells. Cells were stained with commercial rat anti-mVCAM-1 antibody (Millipore, Burlington, MA, USA, Cat# CBL1300, RRID:AB_2214062) as positive control, mIgG_2a_ isotype negative control (Miltenyi Biotec, Bergisch Gladbach, Germany, Cat# 130-106-546, RRID:AB_2661589), or clone 3C12 in mIgG_2a_ format, followed by appropriate secondary antibodies. The table shows the normalized MFI (median fluorescence intensity) values for each of the antibody combinations, with lighter colors indicating lower antibody binding and darker colors indicating higher antibody binding. Displayed is the average normalized MFI (mVCAM-1-eGFP-positive+antibody-positive cells quadrant normalized to MFI of the mVCAM-1-eGFP-negative+antibody-positive cells quadrant) from one experiment with 2 replicates.

**Supplementary Table S1:** Summary of Tukey’s multiple comparisons of non-treated and antibody-treated groups against the lipopolysaccharide (LPS)-stimulated group for spectrophotometric analysis of the static adhesion assay (**Figure 2M**).

| **Comparison** | **Mean difference** | **95.00% CI of diff.** | **Significance** | **Adjusted P-value** |
| --- | --- | --- | --- | --- |
| LPS-stimulated vs. **non-treated** | -37.04 | -51.27 to -22.80 | **** | <0.0001 |
| LPS-stimulated vs. **+VE** mAb | 41.51 | \| 27.27 to 55.74 \| \| --- \| | **** | <0.0001 |
| LPS-stimulated vs. **-VE** mAb | 2.085 | -12.15 to 16.32 | ns | >0.9999 |
| LPS-stimulated vs. **1A9** mAb | 21.70 | 7.462 to 35.94 | *** | 0.0002 |
| LPS-stimulated vs. **2D3** mAb | 16.63 | 2.396 to 30.87 | * | 0.0102 |
| LPS-stimulated vs. **2D8** mAb | 31.98 | 17.74 to 46.22 | **** | <0.0001 |
| LPS-stimulated vs. **2E2** mAb | 39.27 | 25.03 to 53.50 | **** | <0.0001 |
| LPS-stimulated vs. **2E6** mAb | 26.58 | 12.34 to 40.82 | **** | <0.0001 |
| LPS-stimulated vs. **3C12** mAb | 32.97 | 18.73 to 47.21 | **** | <0.0001 |
| LPS-stimulated vs. **3H4** mAb | 29.34 | 15.10 to 43.58 | **** | <0.0001 |

**Supplementary Table S2:** Summary of Tukey’s multiple comparisons of non-treated and antibody-treated groups against the commercial-grade, anti-vascular cell adhesion molecule (VCAM-1) positive control antibody (429)-treated group for spectrophotometric analysis of the static adhesion assay (**Figure 2M**).

| **Comparison** | **Mean difference** | **95.00% CI of diff.** | **Significance** | **Adjusted P-value** |
| --- | --- | --- | --- | --- |
| +VE mAb vs. **non-treated** | 4.470 | -9.768 to 18.71 | ns | 0.9923 |
| +VE mAb vs. **LPS-stimulated** | 41.51 | 27.27 to 55.72 | **** | <0.0001 |
| +VE mAb vs. **-VE** mAb | -39.42 | -53.66 to -25.18 | **** | <0.0001 |
| +VE mAb vs. **1A9** mAb | -19.81 | -34.04 to -5.568 | *** | 0.0009 |
| +VE mAb vs. **2D3** mAb | -24.87 | -39.11 to -10.63 | **** | <0.0001 |
| +VE mAb vs. **2D8** mAb | -9.527 | -23.77 to 4.711 | ns | 0.4835 |
| +VE mAb vs. **2E2** mAb | -2.241 | -16.48 to 12.00 | ns | >0.9999 |
| +VE mAb vs. **2E6** mAb | -14.93 | -29.17 to -0.6904 | * | 0.0324 |
| +VE mAb vs. **3C12** mAb | -8.537 | -22.78 to 5.701 | ns | 0.6409 |
| +VE mAb vs. **3H4** mAb | -12.17 | -26.40 to 2.072 | ns | 0.1604 |
